# Correction to “Tolerability and first hints for potential efficacy of motor‐cognitive training under inspiratory hypoxia in health and neuropsychiatric disorders: A translational viewpoint”

**DOI:** 10.1002/nep3.64

**Published:** 2024-11-28

Mennen et al., Neuroprotection. 2024;2:228–242. DOI:10.1002/nep3.47

Unfortunately, the funding information in both the funding statement and the acknowledgement is not correct. It should read as follows.

**In the funding information (p.229):**

“ERC Advanced Grant acronym BREPOCI, Grant/Award Number: 101054369; ALTIBRAIN, Grant/Award Number: 101043416; DFG, Grant/Award Number: TRR‐ 274/1 2020‐408885537”

Should please be replaced by

“ERC Consolidator Grant to Dr. Miskowiak, acronym: ALTIBRAIN, Grant/Award Number: 101043416; DFG, Grant/Award Number: TRR‐ 274/1 2020‐408885537”

**In the acknowledgements (p.241):**

“This work has been supported by the European Research Council (ERC) Advanced Grant to HE under the European Union's Horizon Europe research and innovation program (acronym BREPOCI; Grant Agreement No. 101054369), as well as an ERC Consolidator Grant to Kamilla Woznica Miskowiak (acronym ALTIBRAIN; Grant Agreement No. 101043416), in collaboration with Hannelore Ehrenreich. Furthermore, the study has been supported by the Max Planck Society and the Max Planck Förderstiftung. Research in the labs of Hannelore Ehrenreich and Klaus‐Armin Nave is funded by DFG TRR‐274/1 2020‐408885537. Klaus‐Armin Nave is supported by the Adelson Medical Research Foundation.”

Should please be replaced by

“This work has been supported by the Max Planck Society and the Max Planck Förderstiftung and an ERC Consolidator Grant to Kamilla Woznica Miskowiak (acronym ALTIBRAIN; Grant Agreement No. 101043416), in collaboration with Hannelore Ehrenreich. Research in the labs of Hannelore Ehrenreich and Klaus‐Armin Nave is funded by DFG TRR‐274/1 2020‐408885537. Furthermore, Klaus‐Armin Nave is supported by the Adelson Medical Research Foundation.”

The authors apologize for these errors.

